# A C-terminal cysteine residue is required for peptide-based inhibition of the NGF/TrkA interaction at nM concentrations: implications for peptide-based analgesics

**DOI:** 10.1038/s41598-018-37585-5

**Published:** 2019-01-30

**Authors:** Andrew J. Poole, Laura Frigotto, Matthew E. Smith, Claudia Baar, Gabriela Ivanova-Berndt, Agnes Jaulent, Catherine Stace, Christopher G. Ullman, Anna V. Hine

**Affiliations:** 1 School of Life and Health Sciences, Aston University, Aston Triangle, Birmingham, B4 7ET UK; 2 Isogenica Ltd., The Mansion, Chesterford Research Park, Little Chesterford, Essex, CB10 1XL UK; 3 Present Address: Immunocore Ltd., 101 Park Drive, Milton Park, Abingdon, Oxon OX14 4RY UK; 4 Present Address: Evonetix Ltd, Suite 6, Science Village, Chesterford Research Park, Little Chesterford, Essex, CB10 1XL UK; 5 Present Address: Inivata Ltd., The Portway Building, Granta Park, Cambridge, CB21 6GS UK; 6 Present Address: BicycleTx Ltd., Building 900, Babraham Research Campus, Cambridge, CB22 3AT UK; 7 Present Address: espeRare, Campus Biotech, Innovation park, Avenue Secheron 15, CH-1202 Geneva, Switzerland; 8 Present Address: Life Sciences Consultant, Highfields Caldecote, Cambridge, CB23 7GB UK; 9 Present Address: Paratopix Ltd., Bishop’s Stortford, Hertfordshire, CM23 5JD UK

## Abstract

Inhibition of the NGF/TrkA interaction presents an interesting alternative to the use of non-steroidal anti-inflammatories and/or opioids for the control of inflammatory, chronic and neuropathic pain. Most prominent of the current approaches to this therapy is the antibody Tanezumab, which is a late-stage development humanized monoclonal antibody that targets NGF. We sought to determine whether peptides might similarly inhibit the NGF/TrkA interaction and so serve as future therapeutic leads. Starting from two peptides that inhibit the NGF/TrkA interaction, we sought to eliminate a cysteine residue close to the C-terminal of both sequences, by an approach of mutagenic analysis and saturation mutagenesis of mutable residues. Elimination of cysteine from a therapeutic lead is desirable to circumvent manufacturing difficulties resulting from oxidation. Our analyses determined that the cysteine residue is not required for NGF binding, but is essential for inhibition of the NGF/TrkA interaction at pharmacologically relevant peptide concentrations. We conclude that a cysteine residue is required within potential peptide-based therapeutic leads and hypothesise that these peptides likely act as dimers, mirroring the dimeric structure of the TrkA receptor.

## Introduction

Nerve growth factor (NGF) was first described over 65 years ago by Levi-Montalcini & Hamburger^1^ and is the founding member of the neurotrophin family, which play a critical role in controlling the development and survival of neuronal populations in the central and peripheral nervous system^2,3^. Neurotrophins act upon two classes of receptors; the high affinity tropomyosin receptor kinases (Trk) receptors, and the low affinity p75 neurotrophic receptor (p75NTR)^4^.

NGF is of interest to the clinical community owing to its involvement in the perception of pain. When NGF binds TrkA on nociceptive neurons, it activates phospholipase C, which results in sensitisation of TRPV1, a non-selective ligand-gated channel that generates the action potential resulting in pain signal transmission^5,6^. NGF also increases TRPV1 expression and its trafficking to the plasma membrane^6,7^.

Since increased levels of NGF have been implicated in a number of pain states, including inflammatory and neuropathic pain^8^, neurogenic overactive bladder and interstitial cystitis^9,10^, prostatitis^11^, asthma^12^ and cancer-induced bone pain^13^, interest has focussed on creating novel analgesics by inhibiting the NGF/TrkA interaction. Accordingly, an anti-TrkA monoclonal antibody was shown to inhibit the perception of pain in both inflammatory and neuropathic pain models^14^. However to date, the most common NGF/TrkA intervention strategies have involved sequestering NGF with monoclonal antibodies^15^. Such administration has shown good analgesic effect in a number of animal models for human disease, such as fracture pain^16^, cancer pain^17^, pancreatic pain^18^ and arthritic joint pain^19^. Of the various anti-NGF antibodies in clinical trials, Tanezumab has probably been the most successful. It is a humanized IgG2 monoclonal antibody that binds with high affinity and specificity to NGF^20^. Although there have been side effect profile concerns, prompting a hold on all clinical trials of anti-NGF antibodies in 2010, Tanezumab was granted fast track status by the FDA in June 2017 and positive results from Phase 3 clinical trials have been announced recently^21^. A recent study has provided preliminary evidence that efficacy and safety of Tanezumab is similar whether delivered intravenously or subcutaneously^22^.

As described above, NGF binds both to TrkA (part of the Trk family of receptor tyrosine kinases) and to p75NTR. The Trk family share sequence homology and their extracellular regions comprise a cysteine rich domain (domain 1), three leucine rich repeats (domain 2), another cysteine rich cluster (domain 3) and two immunoglobulin (Ig)-like domains (domains 4 and 5)^23^. These extracellular domains are linked to an intracellular kinase domain via a single transmembrane helix. A sequence homology of >75% is observed for the intracellular kinase domain^24^ whilst the extracellular domains show a lesser sequence conservation of 50–55%^25^. NGF binds to domain 5 of TrkA both via a ‘specificity patch’ and a ‘conserved patch’^26^. Unsurprisingly, the ‘specificity patch’ of NGF has low sequence homology with other neurotrophins, whereas the ‘conserved patch’ contains residues conserved with other neurotrophins. Interestingly, neither of these patches are involved in p75NTR binding, which occurs via the L1, L3 and L4 loops of NGF^26^, again interacting with two distinct patches in the p75NTR receptor^27–30^.

Whilst the Trk receptors bind specific neurotrophins, p75NTR, a transmembrane glycoprotein, binds all members of the neurotrophin family^31^. The cytoplasmic domain of p75NTR contains a ‘death domain’, consisting of six α helices, that shares sequence homology to those found in other apoptosis inducing factors. Its extracellular domain contains four cysteine rich domains consistent with the tumour necrosis factor receptor family^32^. The cell mediated effect of NGF-p75NTR binding is dependent on the presence or absence of TrkA. If TrkA is absent, NGF-p75NTR binding will lead to apoptosis^33^ whereas NGF- p75NTR binding in the presence of TrkA will increase the binding affinity of NGF to TrkA which in turn promotes survival^34^.

Development of novel anti-NGF peptides or peptide mimetics to inhibit the NGF/TrkA interaction, as an alternative to antibody therapy, would be potentially more cost-effective and/or a shorter acting addition to the existing arsenal of pain-modulating therapies. Indeed, the concept of developing peptide-based analgesia relating to the NGF-TrkA pathway is not new. Peptide-based inhibitors of the NGF-TrkA interaction were first described in 1995, comprising a series of short synthetic peptides cyclised via oxidation of terminal cysteine residues^35^. Later, linear cell-penetrating peptides, synthesised as HIV Tat fusions, were shown to inhibit NGF-stimulated TrkA phosphorylation and to suppress NGF-induced TRPV1 expression in PC12 cells^36^. Subsequently one of those peptide fusions, named IPTRK3, was shown to supress inflammatory pain in rats after local injection^37^ and cancer-induced pain in mice following intraperitoneal injection^38^. Peptidomimetic antagonists of TrkA have also been developed that prevent NGF/TrkA signalling^39,40^. However, recent reviews of pain therapy involving the NGF-TrkA pathway still focus predominantly on antibody-based (or small molecule) intervention, rather than peptidic alternatives^41,42^. Accordingly, we have produced and screened semi-saturated peptide libraries based on two proprietary anti-NGF peptides, that unlike the early cyclic peptides of Saragovi and co-workers^35^, have only a single cysteine residue close to their C-termini. Herein we describe the analysis of those proprietary peptides and our investigations into the possibility of mitigating the requirement for the C-terminal cysteine residues within their sequences altogether. We conclude that in common with both NGF and TrkA, cysteine residues play an essential role within anti-NGF peptides and as such are critical for effective inhibition of the NGF/TrkA interaction.

## Results

### Preliminary analyses of novel, proprietary, anti-NGF peptides

**Figure 1:**
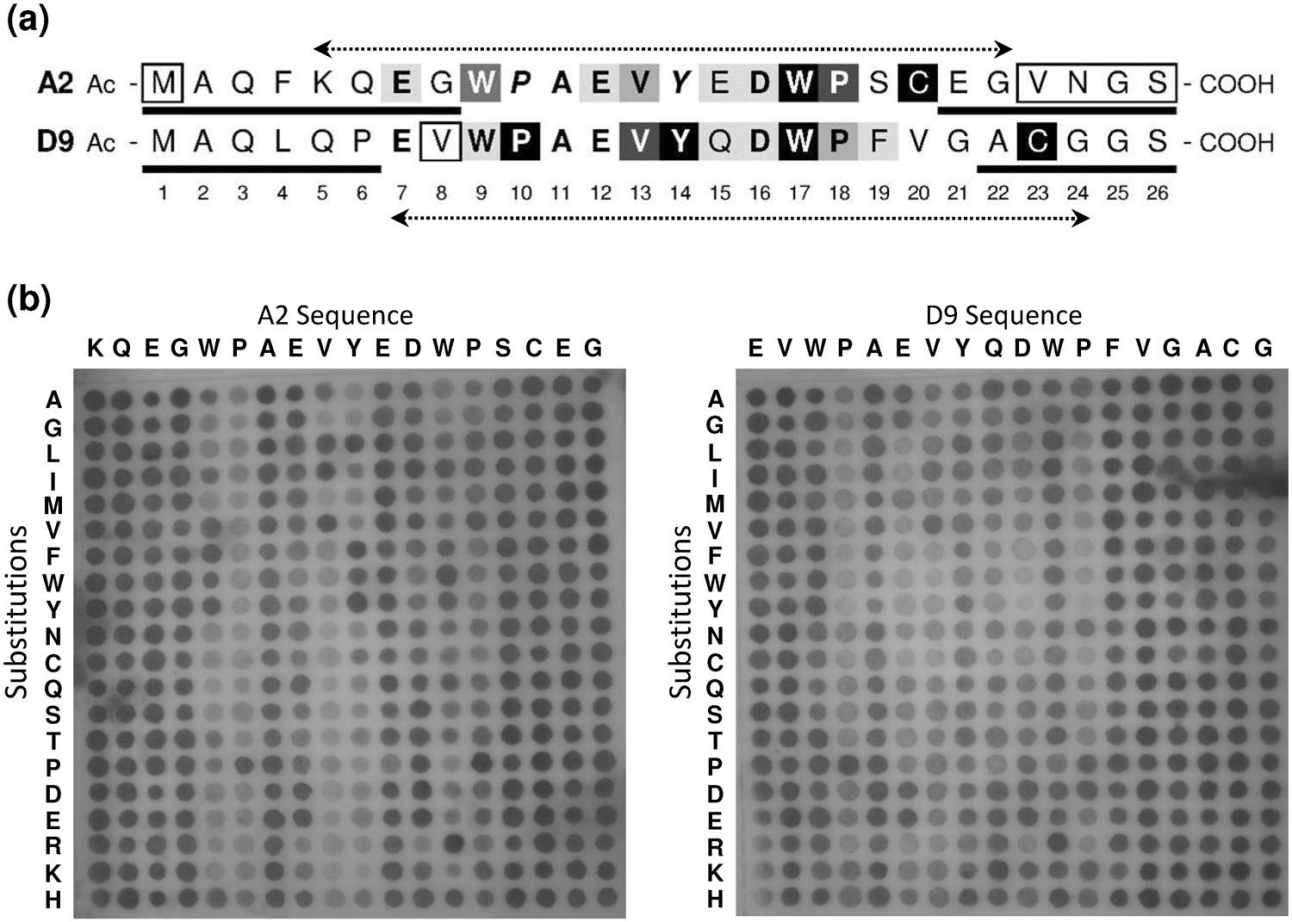
Preliminary analyses of anti-NGF peptides A2 and D9. (**a**) Summary of findings from alanine scanning and deletion analyses. Key - Ala substitution: Back shading indicates the effect of alanine substitution on inhibition with darker shading indicating greater loss of inhibition; italics (peptide A2: positions P_10_ and V_14_) indicates residues not examined by alanine scanning owing to preliminary peptide synthesis failure; boxes indicate single residues in which substitution with alanine leads to a minor increase in inhibition at 20 nM peptide concentration. Source data is illustrated in the Supplementary Fig. 1. Key - deletion analyses: bold underlining indicates residues that may be deleted without loss of inhibition; boxing (peptide A2, COOH terminal) indicates the residues that when collectively deleted, lead to a minor increase in inhibition at 20 nM peptide concentration. Source data is illustrated in the Supplementary Fig. 1. Dotted arrows indicate the 18-mer peptides examined by spot blot analysis (Fig. 1b). All peptides were synthesised in house, by Isogenica Ltd. (**b**) Spot blot analysis. Peptides were spotted onto PVDF membrane and blotted with NGF and anti-NGF antibody. Original peptide sequences are listed across the horizontal axes and substitutions are listed by chemical similarity on the vertical axes. Note that each individual spot represents a single amino acid substitution (identified by reference to the horizontal and vertical axes). Consequently, the parental peptide sequence occurs once in each column and row as repeat internal controls. All peptides were synthesised at Isogenica Ltd. using standard Fmoc chemistry.

Initial studies within Isogenica Ltd had identified two synthetic 26-mer peptides, A2 and D9, that bind nerve growth factor (NGF) and inhibit binding to its receptor, TrkA. Both peptides were predicted to possess partial weakly-helical structure (Supplementary Information, Fig. 1a). Synthetic alanine scans were performed on each peptide and the resulting substituted peptides examined in a competitive ELISA. These analyses suggested that Ala substitution of residues 7, 9, 12, 13, 16–18 and 20 of peptide A2 and residues 9, 10, 13–19 and 23 of peptide D9 diminished the inhibitory activity of each peptide with the effect being most pronounced at positions 9, 17, 18 & 20 and 10, 14, 17 & 23 within peptides A2 and D9 respectively. Meanwhile, substitution of Met_1_ with Ala in peptide A2 and Val_8_ with Ala in peptide D9 each led to a modest increase in inhibition at 20 nM peptide concentrations, whilst Ala substitution at other positions had little or no effect (Supplementary Information, Fig. 1b). Variants of peptides A2 and D9 with truncated terminals were also examined for their ability to inhibit the NGF/TrkA interaction in the competitive ELISA. Removal of up to the first eight or up to the last six residues of peptide A2 had little/no effect on inhibition, whilst deletion of the final four residues 23–26 (in the presence of the residues 1–8) led to a modest increase in inhibition of the NGF/TrkA interaction by peptide A2 at 20 nM concentration. Similarly up to the first six or the last five residues of peptide D9 could be removed without detriment to inhibitory activity, although further deletions at the amino terminal of either peptide led to a reduction in inhibition (Supplementary Information, Fig. 1c). The sequence of peptides A2 and D9 and the results of these preliminary analyses are summarised in Fig. 1a.

To determine residues required for NGF binding within the core of peptides A2 and D9, arrays were synthesised in which each residue within an 18 aa core (Fig. 1a, dotted arrows) was individually substituted with each of the 20 naturally-occurring amino acids. The peptides were then examined by spot blot analysis for their ability to bind NGF. Examination of Fig. 1b largely confirms the findings of the preliminary alanine scan and deletion analyses. For example, Tyr_9_ of peptide A2 and Pro_10_ of peptide D9 are both highly sensitive to substitution. However, a few discrepancies exist between the results of the two assays, most notable of which are the substitutions of Cys_20_ and Cys_23_ (peptides A2 and D9 respectively). Whilst Ala substitution of these residues completely eliminates inhibitory activity (Fig. 1a), in each case the single cysteine residue can be substituted with any of the other 19 amino acids with no apparent effect on NGF binding (Fig. 1b). This difference results from the differing information provided by the two assays. Whilst the preliminary assays examined the peptides’ abilities to inhibit NGF/TrkA interaction, the spot blot simply examines the peptides’ abilities to bind NGF. Taken together, the analyses of parental peptide sequences suggest that the single cysteine residue near to the C terminal of each peptide is required for inhibition of the NGF/TrkA interaction, but is not necessary for NGF binding itself. To examine the role of these cysteine residues, inhibitory activity of DMSO-oxidised samples of the two 18-mer core sequences were compared with unoxidized samples of both the original A2 and D9 26-peptides and their 18-mer cores. All peptides inhibited the NGF-TrkA interaction, but inhibition was enhanced following peptide oxidation (Supplementary Information, Fig. 2), suggesting that the peptides function best as a dimers, presumably mediated via a disulfide bond between cysteine residues close to the C-terminus of each peptide.

### Design and synthesis of an anti-NGF peptide library

The presence of cysteine is undesirable in terms of biological manufacturing and product stability, chiefly owing to its potential for oxidation and poorly-controlled multimerization. The preliminary alanine scanning and dot blot analyses (Fig. 1) had examined the effects of single substitutions on peptide activity. We therefore sought to determine whether an optimal combination of multiply-substituted residues in an anti-NGF peptide could mitigate the requirement for cysteine. If so, a synthetically-optimal anti-NGF peptide could be created.

In designing a peptide library, all three preliminary analyses (alanine scanning, deletion analysis and the substitution array) were taken into account. Both the alanine scan (Supplementary Information, Fig. 1b,c) and the substitution array (Fig. 1b) confirm that individual substitution of residues 1–6 or 19–22 with alanine in either peptide can be achieved without detriment to NGF binding (albeit that substitution of the cysteine residues eliminates inhibitory activity), whilst deletion of residues 23–26 of peptide A2 is mildly advantageous, as described above. Meanwhile, examination of Fig. 1a shows that both peptides A2 and D9 contain a central motif (residues 7–18) in which ten of the twelve amino acids are identical and the substitution array indicates the degree of individual substitutions that can be tolerated at each of these positions.

**Figure 2:**
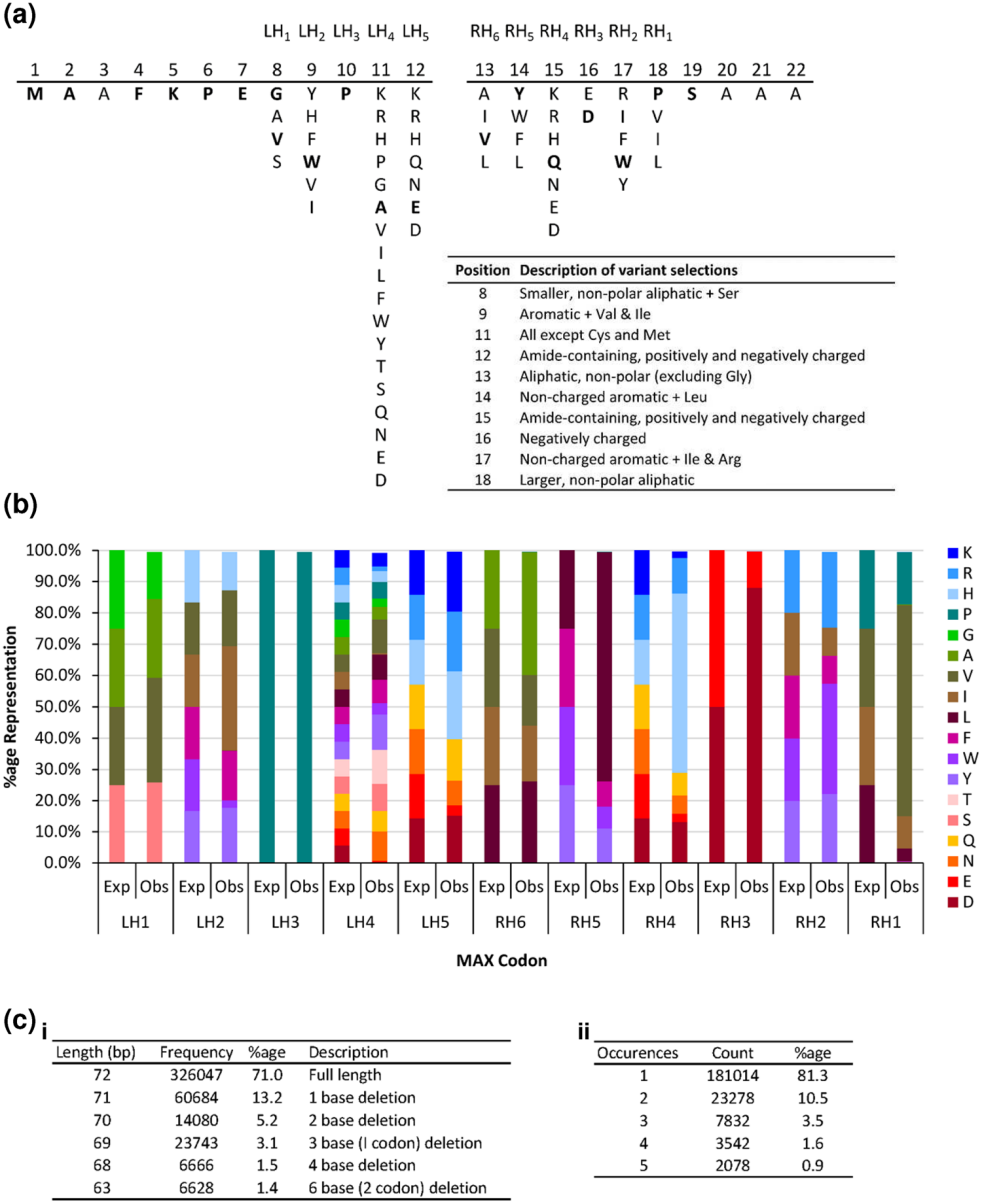
Anti-NGF peptide library design and synthesis. (**a**) Design of anti-NGF peptide library. (**b**) Expected (Exp) *versus* observed (Obs) amino acid frequencies encoded within the anti-NGF gene library. The library was synthesised in two sections (left and right hand sides) with five and six cycles of addition respectively, as described in Materials and Methods, using manual, equimolar additions of MAX hairpin oligonucleotides, followed by blunt-end ligation of the two library fragments. Codon frequencies were determined by Illumina Miseq analysis of full length amplicons (n = 326047 sequences). (**c**) Additional library statistics. (i) Lengths of the most common library products. (ii) Frequencies of repeated sequences within the 326047 full-length library products.

A 22-mer anti-NGF peptide library with conserved residues in positions 1–7, 10 and 19–22 was therefore designed to combine the results of all three analyses. The library was synthesised using ProxiMAX randomisation, in two sections (Fig. 2a) which were subsequently joined by blunt-end ligation. ProxiMAX randomisation is advantageous since, in contrast to conventional saturation mutagenesis, it is a non-degenerate process that allows the user to encode only selected amino acids at each saturated position, thereby creating the smallest possible library while covering all desired mutations. We have previously described ProxiMAX technology and its application via both manual and automated single (trimer) additions and automated double-codon (hexamer) additions^43,44^. Herein, we employed manual, equimolar trimer additions as described in Materials and Methods. The quality of the finished library was assessed by next generation sequencing (Fig. 2b). As previously anticipated, ProxiMAX randomisation via manual, equimolar addition of trimers is not so precise as the automated variant or indeed the manual or automated addition of hexamers. Nonetheless, the resulting library showed acceptable correlation between the expected and observed codon frequencies. The library had a calculated diversity of 68.7%^45^. Further library statistics are illustrated in Fig. 2c.

### Library selection and screening strategy

The major bottleneck in traditional directed evolution is the ability to screen the gene libraries efficiently. In order to screen a large mixture of putative inhibitors such as is created by saturation mutagenesis, a high-throughput assay format is essential. *In vitro*, cell-free selection methods offer greater ability to probe sequence space and when combined with high-quality libraries, maximise the probability of finding optimised mutants. Conversely, the competitive ELISA assay that was used to screen the original alanine-substituted peptides for inhibition of the NGF/TrkA interaction is strictly a low throughput assay that can provide useful data only when used to analyse individual inhibitors. Accordingly, the anti-NGF library was expressed and screened in a three-stage process. The initial screen, using CIS-display^46^ served to enrich a maximal size, oversampled library for mutants retaining NGF binding. In the second-stage, phage display/ELISA were used to eliminate weak and/or non-specific binders. Finally, individual peptides identified from the first two stages were chemically synthesised without C-terminal extensions (RepA or gene III in CIS and phage display formats, respectively) and examined via competitive ELISA for their ability to inhibit the NGF/TrkA interaction. Each of these stages are addressed below.

### Library enrichment through CIS Display

CIS display expresses library genes as fusions to the bacterial replication initiator protein, RepA in an *in vitro* translation and transcription (ITT) system that also includes the DNA elements RepA binds to in CIS. This creates a phenotype/genotype link by exploiting RepA’s ability to bind to its encoding DNA in *cis*^46^. The anti-NGF library (Fig. 2), encoding a theoretical total of 13.5 million variant peptides, was subjected to four rounds of CIS display against an immobilised recombinant human NGF (rhNGF) target, with the stringency of selection conditions progressively increasing through each round, as described in Materials and Methods. The output of the CIS display selections were then inserted into phagemids, yielding approximately 2500 clones.

### Assessment of peptide specificity for NGF via phage display/ELISA

**Figure 3:**
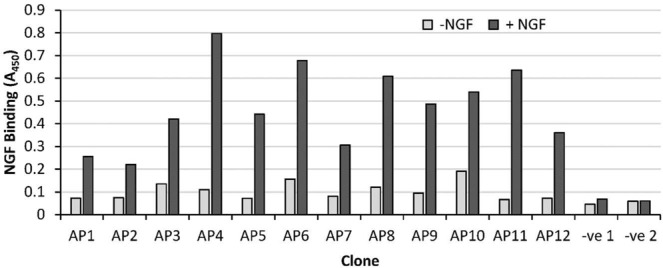
Assessment of peptide specificity for NGF via phage display/ELISA. Two hundred and fifty phage clones were analysed by ELISA for their ability to bind to a microtitre well in the presence (black bars) or absence (grey bars) of immobilised NGF as described in Materials and Methods. Results from the 12 independent, library-compliant, positive clones that showed specific NGF binding are illustrated. Negative controls: −ve 1, non-recombinant phage; −ve 2, PBS buffer control (no phage).

Although increasing in stringency through each round, and despite blocking the microtitre wells, like any panning procedure, the CIS-display protocol has the potential to carry expressed peptides with weak or even non-specific interactions through to the next round of screening. Accordingly, phage display/ELISA was next employed to select specific binders from the enriched library. Approximately 250 clones were chosen and screened via phage display/ELISA for the ability to bind specifically to immobilised rhNGF. Subsequent DNA sequencing of the 32 resulting positive clones revealed 12 twelve independent sequences that were compliant with the library design and exhibited specific NGF binding (Fig. 3). Interestingly, a few frameshifted mutants that contained one or more cysteine residues were also identified amongst the specific peptides. However, since we aimed to eliminate cysteine from the peptide design, only representatives of the twelve compliant sequences were taken forward to the final stage of screening.

### Assessment of peptide-based inhibition of the NGF/TrkA interaction via competitive ELISA

**Figure 4:**
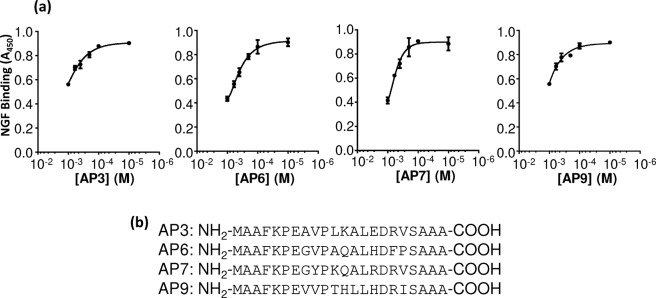
Inhibition of the NGF/TrkA interaction by peptides AP3, AP6, AP7 and AP9. (**a**) Competitive ELISAs. Each of the twelve chemically-synthesised peptides was incubated with NGF and the mixture added to a plate containing immobilised TrkA. After washing, NGF binding to TrkA was detected via a biotinylated, anti-NGF antibody, streptavidin/HRP conjugate and TMP chromogenic reagent, as described in Materials and Methods. Dose response curves are shown for the four peptides, AP3, AP6, AP7 and AP9 that inhibited the NGF-TrkA interaction successfully. All reactions were performed in triplicate. Error bars represent standard deviations. (**b**) Sequences of the inhibitory peptides.

**Figure 5:**
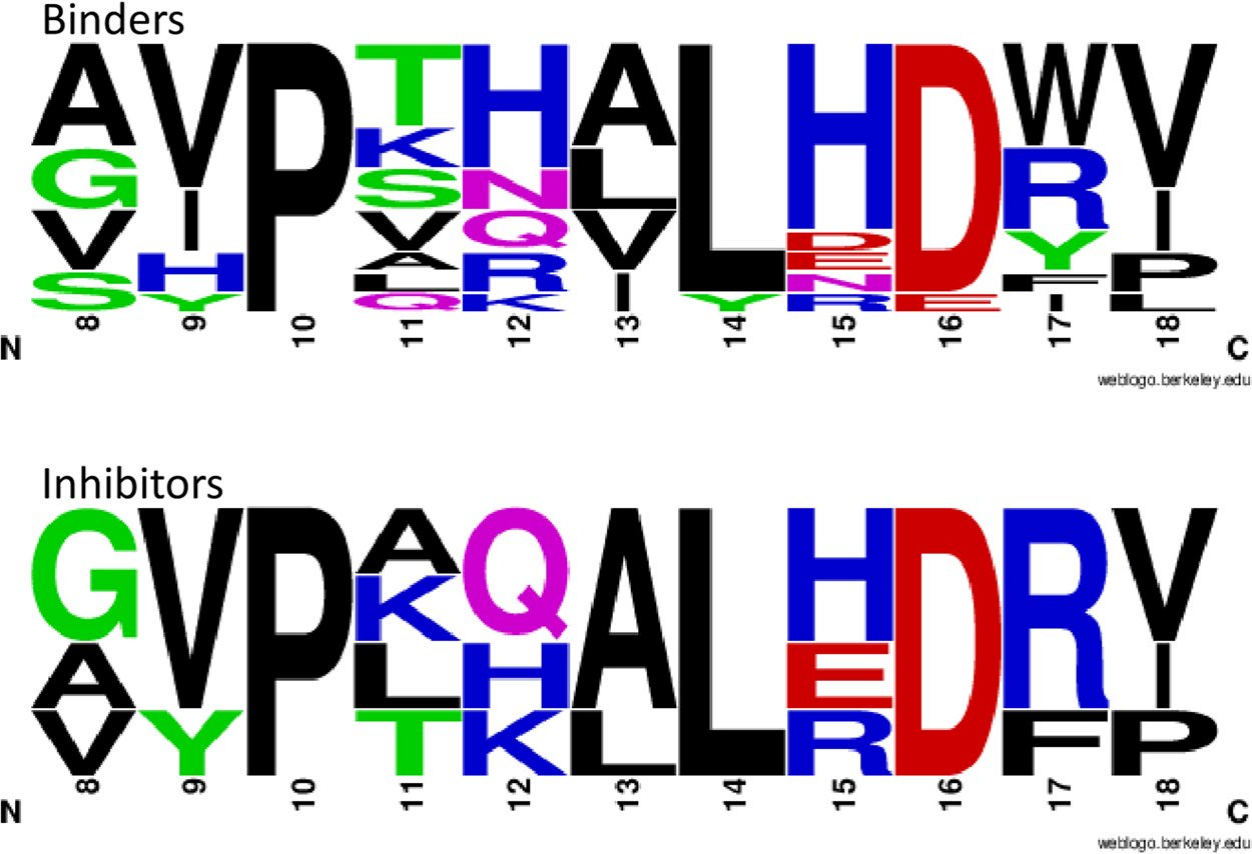
Comparison of non-cysteine-containing peptide sequences selected from anti-NGF library screening that bind NGF, ±inhibition of the NGF/TrkA interaction. Frequency plots illustrate consensus sequences of the saturated region of the twelve NGF-binding peptides assessed in Fig. 3 and the subset of four anti-NGF inhibitory peptides assessed in Fig. 4. Plots were created using the UC Berkeley Weblogo tool (http://weblogo.berkeley.edu/logo.cgi).

The ability to bind NGF specifically is a preliminary assay in the current context, since NGF binding activity does not necessarily translate into the ability to inhibit the NGF/TrkA interaction. Therefore, each unique peptide sequence was chemically synthesised and assessed for its inhibitory activity via competitive ELISA. Interestingly, whereas the inhibitory concentration of the original, cysteine-containing peptides was in the nanomolar range, with full inhibition of the NGF/TrkA interaction being achieved at 100 nM concentrations (Supplementary Information, Fig. 1), none of the selected, cysteine-free peptides showed any inhibition at these concentrations (data not shown). Wider-ranging dose curves from μM to mM concentrations were therefore prepared for each of these peptides. As shown in Fig. 4, four peptides inhibited the NGF/TrkA interaction partially, albeit at these higher concentrations. A comparison between the sequences of the 12 NGF-binding peptides and the subset of four peptides that both bind NGF and inhibit the NGF/TrkA interaction (albeit at elevated concentrations) is illustrated in Fig. 5.

**Figure 6:**
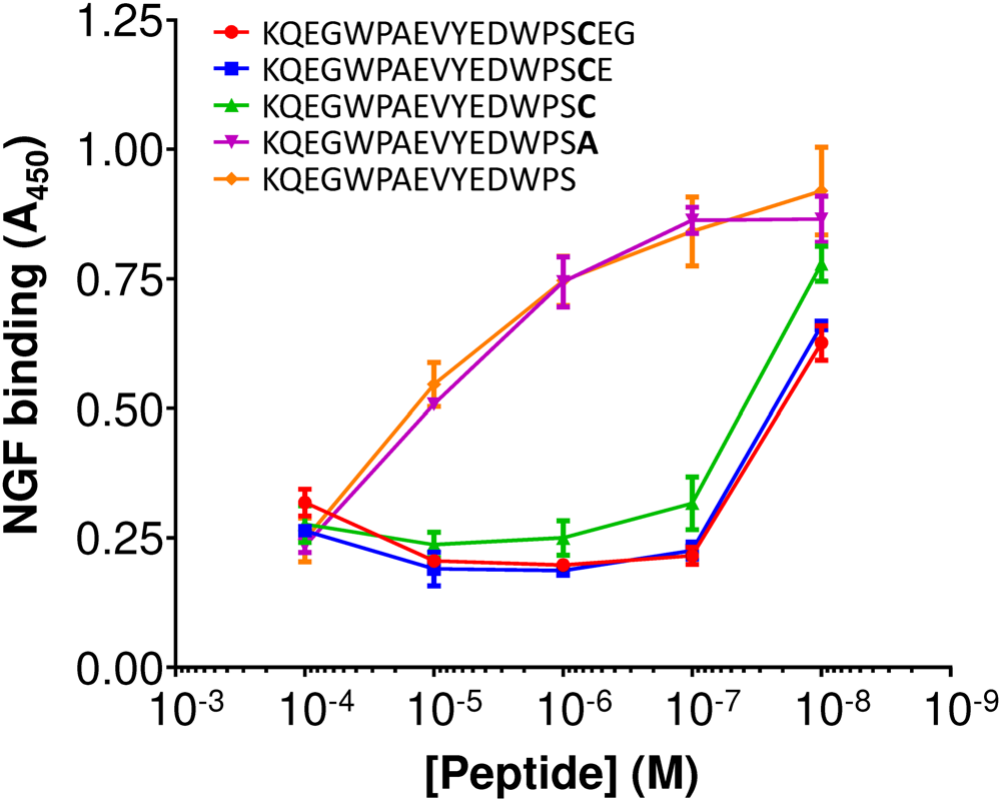
The effect of C-terminal truncation of peptide A2 on the inhibition of the NGF/TrkA interaction. Competitive ELISAs. Each peptide was incubated with NGF and the mixture added to a plate containing immobilised TrkA. After washing, NGF binding to TrkA was detected via a biotinylated, anti-NGF antibody, streptavidin/HRP conjugate and TMP chromogenic reagent, as described in Methods. The sequences of each truncated peptide are shown. All reactions were performed in triplicate. Error bars represent standard deviations.

The far higher effective concentration required for inhibition by the selected peptides, coupled with the re-emergence of cysteine-containing peptides from the second stage screen, suggests that even multiple mutations cannot substitute for a C-terminal cysteine residue and that the latter is indeed required within a peptide in order to inhibit the interaction between NGF and TrkA at reasonable peptide concentrations. To confirm this hypothesis, deletion studies of the core 18-mer of peptide A2 (Fig. 1b) were performed. The cysteine residue is three amino acids away from the C-terminal of this peptide. Therefore the core A2 18-mer and consecutive single amino acid C-terminal deletions were synthesised to encompass the cysteine residue. Dose curves were then constructed using the NGF-TrkA inhibition ELISA described previously. The effect of the cysteine residue is dramatic. In fact, truncation of this peptide has little/no effect on inhibition until the cysteine is removed (Fig. 6). Moreover, lack of inhibition is not merely an effect of truncation: peptide A2-18-2 (Fig. 6, green) which has a cysteine residue at the extreme C-terminus is almost as effective in inhibiting the NGF/TrkA interaction as the core A2 18-mer itself (Fig. 6, red). Conversely, replacement of that cysteine with alanine, even though the peptide is of the same length, or truncation of the terminal cysteine residue, each reduce inhibition of the NGF/TrkA interaction by three orders of magnitude (Fig. 6, magenta and yellow respectively).

## Discussion

Like most other members of neurotrophin family, including brain-derived neurotrophic factor (BDNF)^26,47^, neurotrophin-3 (NT-3)^26,48^, neurotrophin-4/5 (NT-4/5)^26,49^ and neurotrophin-6 (NT-6)^26,50^, biologically active (beta) NGF exists as a parallel homo-dimer of two NGF monomers^26^. The NGF dimer binds two molecules of TrkA by making multiple contacts with the extracellular, Ig-like domain 5 of TrkA (TrkA d5) via two specific areas. Residues 2–13 at the N-terminal of NGF bind to the ‘specificity patch’ of TrkA, which comprises the face of the ABED β-sheet of TrkA d5^26^. Interestingly, in the unbound state, these N-terminal residues of NGF are disordered, but form a short segment of α-helix when bound to the specificity patch of TrkA. The importance of this interaction for the activity of NGF is demonstrated by deletion of the first ten N-terminal residues of NGF, which causes a 300-fold drop in affinity for TrkA^47–51^, whilst a 9-fold decrease in affinity is caused by deletion of the first five residues^48–52^. Thus it is tempting to speculate that anti-NGF peptides might interact with these N-terminal residues of NGF and in so doing, prevent their interaction with TrkA d5. Meanwhile, residues in the central β sheet region or ‘waist’ of NGF bind to the ‘conserved patch’ of TrkA d5, which comprises the AB, C’D loops and particularly the EF loop of TrkA d5 which are all found at one end of TrkA d5 domain^49–53^. It is in this EF loop in which the highest degree of conservation between the Trk receptors is found, with 9 of 11 residues conserved across all three receptors TrkA, TrkB and TrkC^50–54^. As such, inhibition of that interaction will likely be undesirable.

Clearly, in the absence of structural data, the mechanism by which parental peptides A2 and D9 inhibit the NGF/TrkA interaction cannot be elucidated fully. Either peptide might prevent binding between the N-terminal of NGF with the specificity patch of TrkA, or the ‘waist’ of NGF with the conserved patch of TrkA (or even, potentially, both interactions). However, the decreased ability by the non-cysteine-containing peptides isolated in this study to inhibit the NGF/TrkA interaction, compared to the cysteine-containing parental peptides, argue strongly in support of the original hypothesis, namely that the inhibitory peptide itself ideally acts as a dimer. This hypothesis is further supported by the enrichment of cysteine-containing frameshift mutants in the display assays and is logical in terms of the homo-dimeric structure of NGF itself.

However, our results have also demonstrated clearly that the C-terminal cysteine is not required for NGF binding per se. Thus an alternative explanation for inhibition also exists with respect to NGF structure. Each NGF monomer (118 amino acids) contains two pairs of twisted, anti-parallel β- strands. Two such monomers form a dumbbell-shaped homodimer. At the bottom, membrane-facing end of the dumbbell, there are three β-hairpin loop structures (L1, L2 and L4), whilst the ‘waist’ is formed by the central β-sheets of the NGF homo-dimer^26^. Meanwhile, at the top of the dumbbell are both the N- and C- terminals, a β-hairpin loop structure (L3) and a cysteine knot. The latter comprises three disulfide bonds, two of which form a closed ring with the third penetrating the ring. These confer stability to the molecule and interestingly have been found in other dimeric growth factors, such as platelet-derived growth factor and transforming growth factor-β, which do not share any other significant sequence homology with the neurotrophin family^26^. Once the inhibitory peptide has bound NGF via the majority of its residues, might the single cysteine at the C-terminus of the peptide interfere with the cysteine knot, either via labile redox chemistry, metal chelation effects or even a formal disulfide exchange reaction? Any such modification would be reversible but would likely effect both the structure and stability of NGF. The data, namely that peptides lacking a C-terminal cysteine can nevertheless inhibit the NGF/TrkA interaction (albeit at elevated concentrations), argue against such a model, but clearly, future structural data will be required to resolve this question unequivocally.

## Conclusions

Our data confirm that several related peptide sequences can bind to NGF and that NGF-binding does not necessarily equate to inhibition of the NGF/TrkA interaction. Conversely, we conclude that a cysteine residue located close to the C-terminal of a binding peptide is critical for effective inhibition of the NGF/TrkA interaction, but is not itself required for NGF binding. Further, we demonstrate that both cysteine-containing peptide monomers and dimers inhibit the NGF/TrkA interaction, but that inhibition is enhanced in the presence of the dimeric forms of the peptides, relative to the monomeric forms.

Future structural studies will be required to confirm the mode of action of the peptides described herein and indeed any other peptide-based leads. Clearly, an effective peptide-based inhibitor would be required to inhibit the interaction between the N-terminal of NGF and the specificity patch of TrkA, rather than that between the waist of NGF and the conserved patch of TrkA, since the latter shares a high degree of homology with other Trk receptors.

In the meantime, those seeking to develop non-antibody, lead compounds for pain relief via the NGF/TrkA pathway may need to compromise manufacturing ideals and include a C-terminal cysteine residue into peptide-based inhibitors.

## Methods

### ProxiMAX Randomised Anti-NGF Peptide Library Construction

Appropriate self-hybridised MAX hairpin stocks^43^ (2.4 µM) were ligated individually to 0.4 µM base sequence, in a 20 µl reaction containing 1 Thermo Scientific ligase buffer, 5% w/v PEG (Thermo Scientific) and 5 µl *T4 DNA ligase* (Thermo Scientific) and incubated at 22 °C for 15 min followed by 65 °C for 10 minutes.

Quadruple 100 µl amplifications were set up containing 50 pmol primers (the appropriate cycle primer and a universal primer for the base sequence), 20 µM dNTPs, 2 units *Phusion Hot Start II* high-fidelity DNA polymerase (Thermo Scientific), 1 × Phusion HF buffer and 8 µl of the single hairpin ligation. These underwent initial denaturation at 98 °C for 45 secs followed by 3 cycles of (98 °C, 10 s; 52 °C, 30 s; 72 °C, 20 s), 13 cycles of (98 °C, 10 s; 55 °C, 30 s; 72 °C, 20 s) and a final extension of 72 °C for 8 min. The four PCR reactions were combined and purified using a QIAquick PCR Purification Kit (Qiagen) according to manufacturer’s instructions. DNA concentration was quantified by UV using a nanodrop 2000c (Thermo Scientific), PCR products were then mixed to give an equimolar mix of MAX hairpins.

The equimolar mix was digested in a 50 µl reaction containing 1x fast digest buffer and 10 units *Mly*I (Thermo Scientific). The reaction was incubated at 37 °C for 1 hr and then 65 °C for 20 min, and the resulting product was electrophoresed of a 3% agarose/TAE gel to check the digestion was successful and purified by agarose gel extraction. The concentration of the digested product was quantified by UV. This process was then reiterated using the digested material as the template sequence for the following cycle of additions.

### CIS display Selections

The ProxiMAX generated peptide library was then extended on 5′ and 3′ ends with promotor and *RepA*/CIS/*ori* regions encoding DNA, respectively^46^. Next, an *in vitro* transcription/translation reaction (ITT) was set up containing 2 μg library template DNA along with in Isogenica proprietary buffer, amino acid mix and cell lysate to a total volume of 50 μl, and incubated for 1 hour at 37 °C and on ice for 5 minutes. Dynabeads® M-280 Streptavidin (Life technologies) were prewashed 3x with PBS and resuspended in the original volume of 2% BSA/PBS/0.1%Tween (PBST). Non-specific binders were deselected by mixing the ITT for 30 minutes with 2% BSA/PBS (+/−1 mg/ml heparin depending on stringency) and uncoated magnetic beads. These magnetic beads were then removed using a magnet, yielding deselected ITT supernatant. In parallel, biotinylated rhβ-NGF (10 μg) was bound to 50 μl prewashed beads for 1 hour in PBS followed by 2x PBS washes and blocked with 2% BSA/PBS. Deselected ITT supernatant was mixed with rhβ-NGF precoated beads and washed on a KingFisher™ Flex Magnetic Particle Processor (Thermo Scientific). Bead-bound DNA was heat eluted at 75 °C in 1x ThermoPol buffer for 10 minutes, the beads were captured and the supernatant transferred to a fresh Eppendorf tube.

Eluted DNA was PCR amplified using reactions containing 10 μM recovery primer, 2 µM dNTPs, 1 x KOD buffer, 25 mM MgSO_4_, 0.5 units KOD Hot Start DNA polymerase (Merck Millipore Millipore) and 10 µl of the eluted DNA. These underwent initial denaturation at 95 °C for 2 min followed by 35 cycles of (95 °C, 30 s; 52 °C, 20 s; 72 °C, 1 min), and a final extension of 72 °C for 7 min. The resulting product was purified using Promega Wizard^TM^ columns according to the manufacturer’s protocol and the purified DNA was used for another round of selections.

### Peptide dot blot analysis

Peptides were synthesised in house at Isogenica and spotted on a PVDF membrane, which was air-dried and then blocked by incubation in 0.1% casein, 4% sucrose, 0.1% Tween at 4 °C, overnight. The membrane was washed with TBS/tween (3 × 5 min) and TBS (2 × 5 min) and incubated with NGF (3 ng/µl in 0.1% casein/TBS/Tween) at room temperature for 1 hr. The washing steps were repeated and the membrane incubated with 0.025 ng/µL of biotinylated anti-human b-NGF antibody in 0.1% casein/TBS/Tween at room temperature for 1 hr. The washing steps were again repeated and immobilised antibody was then detected using a Strep-Tactin AP detection kit (IBA Lifesciences, cat# 2-1503-000) according to manufacturer’s instructions. All incubations were performed on a rocking platform.

### Phage Screening of CIS Display Selection Output

Final selection round output DNA was then ligated into a standard phagemid vector and transfected into *E. coli*. Transfected *E. coli* were plated in selective medium. Individual colonies were then picked into 120 μl 2TY with 100 μg/ml ampicillin and 2% glucose. This was incubated at 37 °C, 225 rpm until cloudy. Glycerol was then added to 25% (v/v) and these precultures were stored at −80 °C.

Preculture (30 μl) was transferred to 300 μl 2TY-Amp-Glu and incubated at 37 °C until the OD_600nm_ was 0.3. Approximately 10^8^ pfu (plaque forming units) of M13K07 helper phage (NEB) was added and incubated at 37 °C with shaking at 225 rpm. The cultures were pelleted by centrifugation at 3000 × g for 10 mins, the supernatant discarded and pellets resuspended in 600 μl 2TY-Amp. This was subsequently incubated at 37 °C, with shaking at 225 rpm overnight and the following day the cultures were pelleted by centrifugation at 3000 × g for 10 mins. Phage-containing supernatant was analysed by ELISA.

A NUNC F96 Maxisorp plate (Sigma-Aldrich) was coated with 50 ng/well recombinant human β-NGF (R&D Systems) and incubated overnight at 4 °C. The plate was washed 4x PBST and 2x PBS and blocked for 1 hr at room temperature with Blocker^TM^ 1% casein in PBS (Thermo Scientific #37528) followed by 4x washes with PBST and 2x PBS. Supernatant produced from phage expression was diluted 1/2 in 0.1% casein in PBS/0.1% Tween, 100 μl/well was added to the Maxisorp plate and incubated at room temperature for 1 hour. The plate was washed with 4x PBST and 2x PBS. Mouse anti-M13 HRP conjugated antibody (Sigma-Aldrich; 1:5000 dilution, 50 µl/well) in 0.1% casein/PBST was incubated on the plate for 30 minutes at room temperature followed by 4x washes with PBST and 2x PBS. TMB reagent (Thermo Scientific #34028; 50 µl/well) was incubated on the plate for 5–20 minutes, the reaction was quenched with 50 µl/well 0.5 M H_2_SO_4_ and the absorbance read at 450 nm.

### Competitive ELISA analysis of synthetic peptides

Recombinant human TrkA-Fc chimera (125 ng/50 μl; R&D Systems, 175-TK) was coated onto NUNC F96 Maxisorp ELISA plates (Sigma-Aldrich) at 4 °C overnight. The plate was washed 3x in PBST and 2x PBS and then blocked for 1 hr at room temperature with Blocker^TM^ 1% casein in PBS (Thermo Scientific #37528), followed by 4x washes with PBST and 2x PBS. Thirty ng recombinant human β-NGF (R&D Systems, 256-GF/CF) was mixed with 50 µl peptide (Isogenica or Alta Biosciences, UK) at appropriate concentration in 0.1% casein in PBS/0.1% Tween. NGF/peptide solutions (55 µl/well) were transferred into the NUNC Maxisorp plate and incubated for 40 minutes at room temperature with 50 µl/well 1:200 diluted biotinylated anti-human β-NGF antibody (R&D Systems, 256-GF/CF) in 0.1% casein in PBS/0.1% Tween. The plate was washed 4x in PBST and 2x PBS. Streptavidin-HRP (Sigma-Aldrich; Streptavidin-Peroxidase Polymer, Ultrasensitive, #2438; 1:1000 dilution, 50 µl/well) in 0.1% casein/PBST was incubated on the plate for 30 minutes at room temperature followed by 4x washes with PBST and 2x PBS. TMB reagent (Thermo Scientific #34028; 50 µl/well) was incubated on the plate for 5–20 minutes, quenched with 50 µl/well 0.5 M H_2_SO_4_ and the absorbance read at 450 nm.

## Supplementary information

Supplementary Information

Source data

## Data Availability

Source data for Fig. 1 is presented in Supplementary Fig. 1. Source data for Supplementary Figs 1 and 2 and Figs 2–4 and 6 are available in the Aston Data Explorer and may be accessed via the following link 10.17036/researchdata.aston.ac.uk.00000375. Source data for Fig. 5 is contained within the manuscript. Other datasets generated and/or analysed during the current study are available in the Aston Research Explorer and may be accessed via the following link: http://publications.aston.ac.uk/28900/1/Poole_Andrew_J._2016.pdf.
