# Supplementary material for: A C-terminal cysteine residue is required for peptide-based inhibition of the NGF/TrkA interaction at nM concentrations: implications for peptide-based analgesics
Source: Sci Rep. 2019 Jan 30;9:930. doi: 10.1038/s41598-018-37585-5 (PMC6353895; doi:10.1038/s41598-018-37585-5)
Supplement: Supplementary file 1 — Supplementary Information [file 41598_2018_37585_MOESM1_ESM.pdf]

A C-terminal cysteine residue is required for peptide-based inhibition of the NGF/TrkA interaction at nM concentrations: implications for peptide-based analgesics.

Andrew J. Poole[1,†], Laura Frigotto[2,‡], Matthew E. Smith[2,§], Claudia Baar[2], Gabriela Ivanova-Berndt[2,§§], Agnes Jaulent[2,¶], Catherine Stace[2,•], Christopher G. Ullman[2,°] and Anna V. Hine[1,*]

Supplementary Figure 1

(a)

A2:

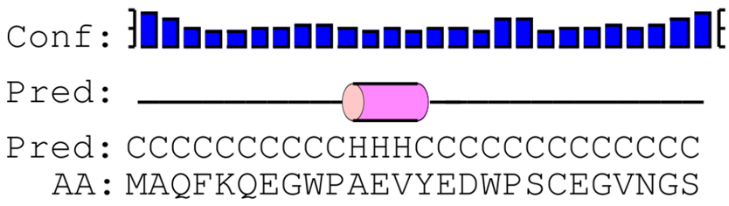


```
Sequence    : MAQFKQEGWPAEVYEDWPSCEGVNGS
Structure   : CCCCCCCCCCHHHCCCCCCCCCCCCC
Helix Prob  : 00123220013432121011210000
Sheet Prob  : 00110000000010100010002100
Coil  Prob  : 96433445542224436634454569
```

D9

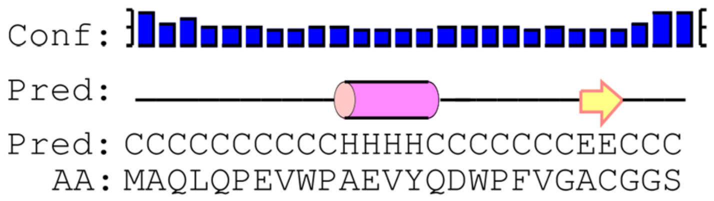


```
Sequence    : MAQLQPEVWPAEVYQDWPFVGACGGS
Structure   : CCCCCCCCCCHHHHCCCCCCCEECCC
Helix Prob  : 00012132113333011121100000
Sheet Prob  : 01010001100000110011133200
Coil  Prob  : 85644433442323444434322589
```

(b)

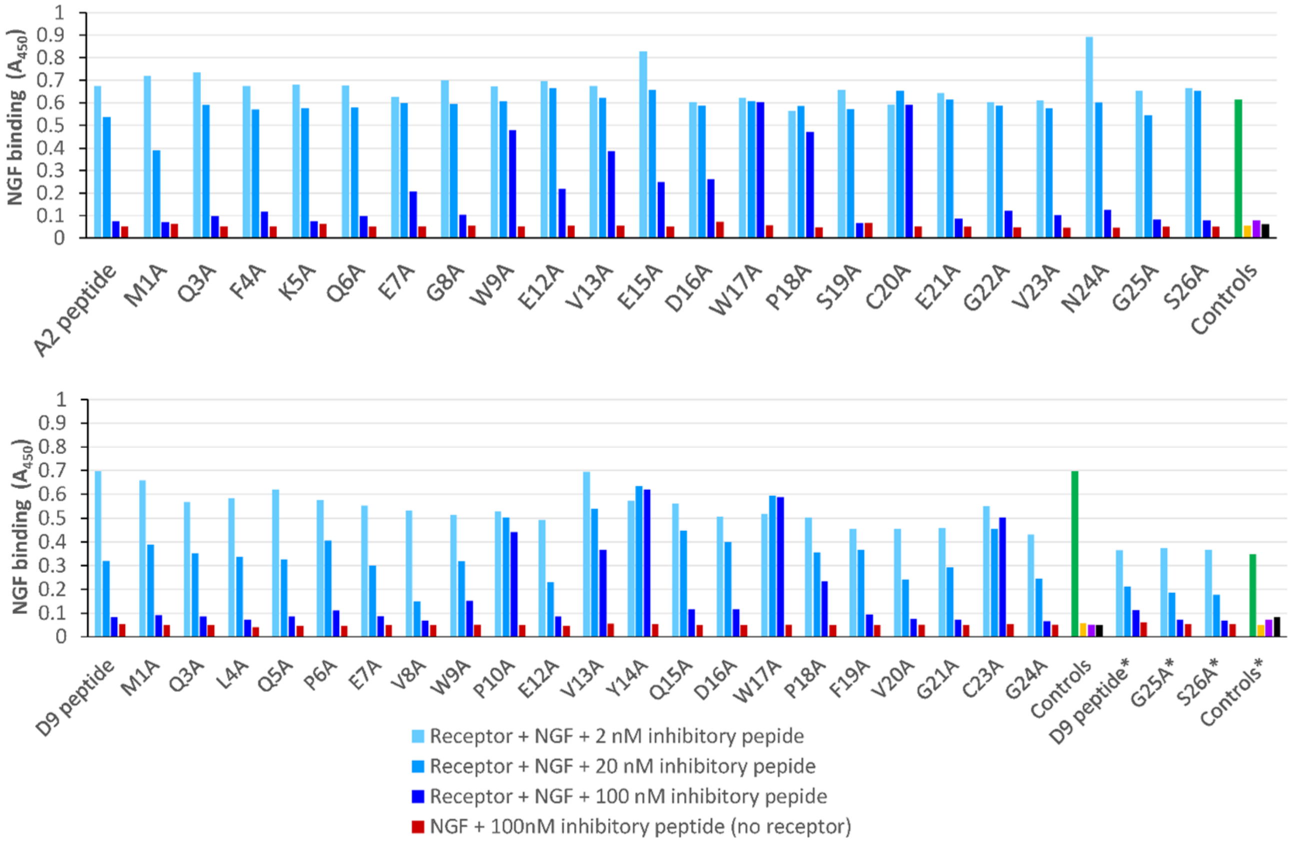
NGF binding (A450)
A2 peptide
M1A
Q3A
F4A
K5A
Q6A
E7A
G8A
W9A
E12A
V13A
E15A
D16A
W17A
P18A
S19A
C20A
E21A
G22A
V23A
N24A
G25A
S26A
Controls
D9 peptide
L4A
Q5A
P6A
V8A
P10A
Y14A
Q15A
F19A
V20A
G21A
C23A
G24A
D9 peptide*
G25A*
S26A*
Controls*
Receptor + NGF + 2 nM inhibitory pepide
Receptor + NGF + 20 nM inhibitory pepide
Receptor + NGF + 100 nM inhibitory pepide
NGF + 100nM inhibitory peptide (no receptor)

(c)

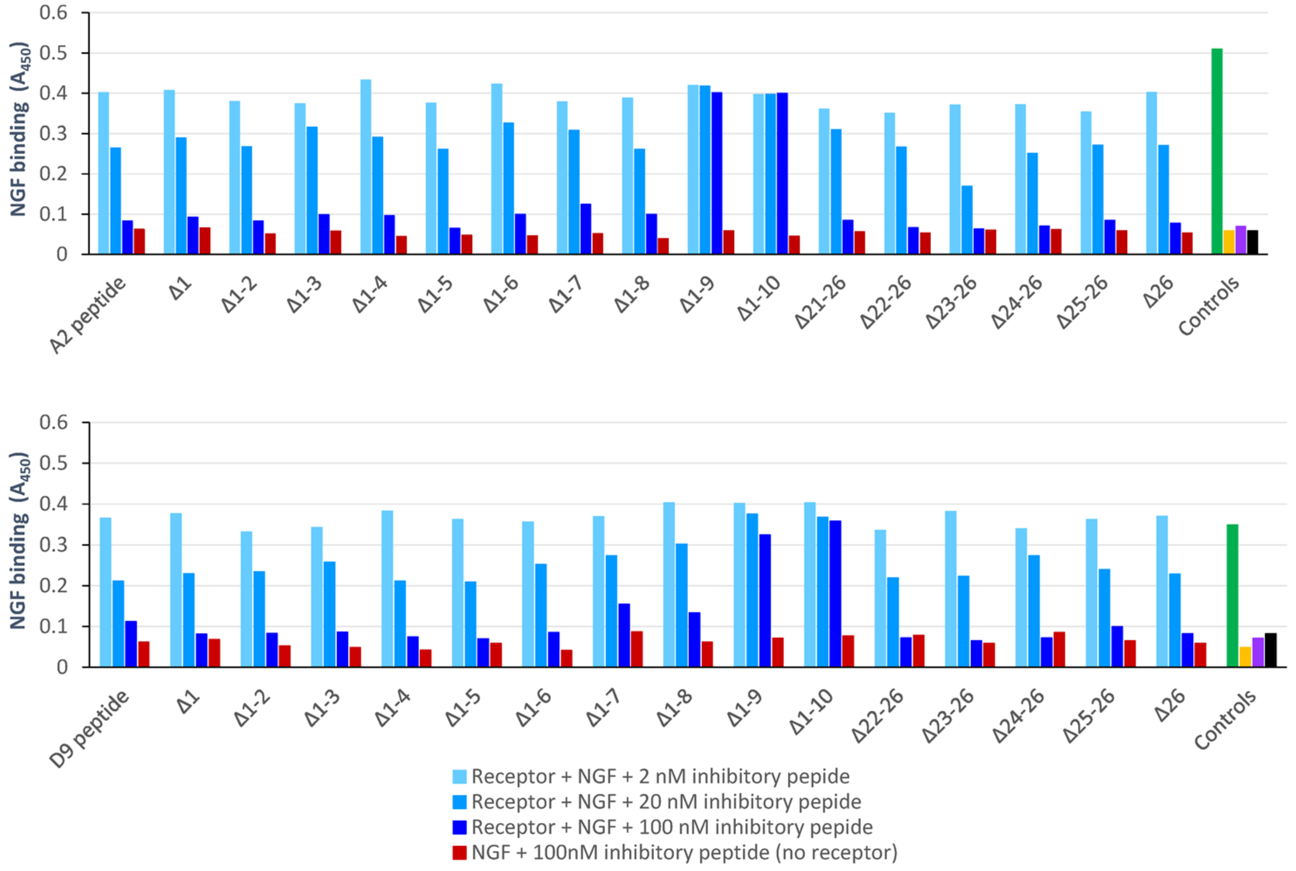
NGF binding (A450)
A2 peptide
Δ1
Δ1-2
Δ1-3
Δ1-4
Δ1-5
Δ1-6
Δ1-7
Δ1-8
Δ1-9
Δ1-10
Δ21-26
Δ22-26
Δ23-26
Δ24-26
Δ25-26
Δ26
Controls
D9 peptide
Receptor + NGF + 2 nM inhibitory pepide
Receptor + NGF + 20 nM inhibitory pepide
Receptor + NGF + 100 nM inhibitory pepide
NGF + 100nM inhibitory peptide (no receptor)

Supplementary Figure 1: Preliminary analysis of peptides A2 and D9.

(a) Helical Prediction of A2 and D9 Peptide Sequences. The propensity for helicity was assessed using the PEP2D webservice for predicting secondary structure of peptides, http://crdd.osdd.net/raghava/pep2d/submit.html. Helix (H), sheet (E) and coil (C) propensities were scored. A weak propensity for helix in both peptides was predicted.

(b) Alanine-substituted peptides as indicated. **(c)** Deleted peptides as indicated.
(b) & (c) Chemically-synthesised peptides were incubated with NGF and the mixture added to a plate containing immobilised TrkA. After washing, NGF binding to TrkA was detected via a biotinylated, anti-NGF antibody, streptavidin/HRP conjugate and TMP chromogenic reagent, as described in Methods. Controls: Green, Receptor + NGF (no inhibitory peptide); Yellow, NGF only (no receptor or inhibitory peptide); Purple, Receptor only (no NGF or peptide); Black, -ve (PBS only, no receptor, NGF, or peptide). *Peptides analysed by separate experiment.

Supplementary Figure 2

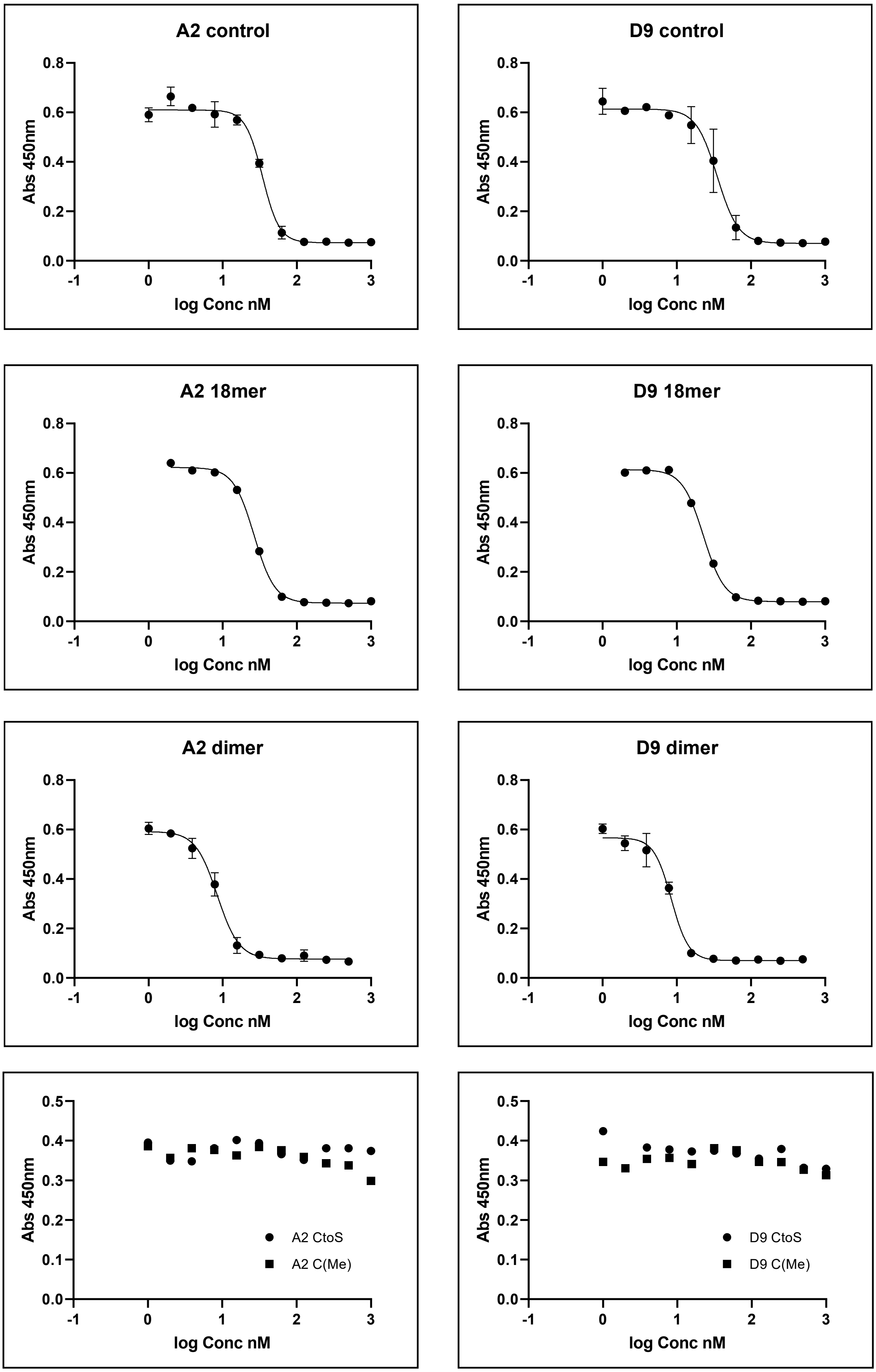

Supplementary Figure 2: The effect of peptide oxidation on inhibition of the NGF-TrkA interaction.

The sequences of peptides A2, D9 and their respective core 18 mers are illustrated in Figure 1. In peptides designated CtoS and CMe, the cysteine residue was replaced with serine and methyl cysteine residues respectively. (Homo)dimers of the A2 and D9 18mer peptides were obtained by DMSO oxidation, followed by purification as described previously[1]. Concentrations of peptide solutions were measured in triplicate, using ε_{280nm}= 12,490 $M^{-1}.cm^{-1}$ for monomers and ε_{280nm}= 25,105 $M^{-1}.cm^{-1}$ for dimers. Each binding experiment was obtained from a freshly prepared solution of purified peptide in ACN:H_2O (1:1). Prior to individual assays, MS and analytical HPLC of the peptide solutions were taken to confirm the identity and purities of the peptides, and also to confirm the lack of dimers in the monomeric peptide solutions. The D9 peptide series was in general less soluble than the A2 series with some D9 peptides precipitating over time (as measured from the decrease in 280nm absorbance). Consequently, all peptide solutions were centrifuged prior to reading the concentration of resulting supernatants. NGF-TrkA inhibition was assayed by competitive ELISA analysis as described in Methods in duplicate or triplicate analyses (according to peptide availability) as detailed in Source Data (https://doi.org/10.17036/researchdata.aston.ac.uk.00000375), with the exception of the substituted CtoS and CMe peptides, which were each analysed in single experiments.

Reference

1. Tam, J.P., Wu, C.-R., Liu, W. & Zhang, J.-W. Disulfide bond formation in peptides by dimethyl sulfoxide. Scope and applications. *J. Am. Chem. Soc.* **113**, 6657-6662 (1991).
